# Measuring adherence to antiretroviral treatment in resource-poor settings: The feasibility of collecting routine data for key indicators

**DOI:** 10.1186/1472-6963-10-43

**Published:** 2010-02-19

**Authors:** John C Chalker, Tenaw Andualem, Lillian N Gitau, Joseph Ntaganira, Celestino Obua, Hailu Tadeg, Paul Waako, Dennis Ross-Degnan, INRUD-IAA

**Affiliations:** 1 Center for Pharmaceutical Management, Management Sciences for Health, Arlington Virginia, USA; 2 Management Sciences for Health, Addis Ababa, Ethiopia; 3 Management Sciences for Health/INRUD, Nairobi, Kenya; 4 Department of Epidemiology and Biostatistics, School of Public Health, National University of Rwanda; 5 Department Pharmacology and Therapeutics, Makerere University Medical School, Kampala, Uganda; 6 Harvard Medical School and Harvard Pilgrim Health Care, Boston, MA, USA; 7 The International Network for the Rational Use of Drugs Initiative on Adherence to Antiretrovirals, Arlington, VA 22203, USA

## Abstract

**Background:**

An East African survey showed that among the few health facilities that measured adherence to antiretroviral therapy, practices and definitions varied widely. We evaluated the feasibility of collecting routine data to standardize adherence measurement using a draft set of indicators.

**Methods:**

Targeting 20 facilities each in Ethiopia, Kenya, Rwanda, and Uganda, in each facility we interviewed up to 30 patients, examined 100 patient records, and interviewed staff.

**Results:**

In 78 facilities, we interviewed a total of 1,631 patients and reviewed 8,282 records. Difficulties in retrieving records prevented data collection in two facilities. Overall, 94.2% of patients reported perfect adherence; dispensed medicine covered 91.1% of days in a six month retrospective period; 13.7% of patients had a gap of more than 30 days in their dispensed medication; 75.8% of patients attended clinic on or before the date of their next appointment; and 87.1% of patients attended within 3 days.

In each of the four countries, the facility-specific median indicators ranged from: 97%-100% for perfect self-reported adherence, 90%-95% of days covered by dispensed medicines, 2%-19% of patients with treatment gaps of 30 days or more, and 72%-91% of appointments attended on time. Individual facilities varied considerably.

The percentages of days covered by dispensed medicine, patients with more than 95% of days covered, and patients with a gap of 30 days or more were all significantly correlated with the percentages of patients who attended their appointments on time, within 3 days, or within 30 days of their appointment. Self reported recent adherence in exit interviews was significantly correlated only with the percentage of patients who attended within 3 days of their appointment.

**Conclusions:**

Field tests showed that data to measure adherence can be collected systematically from health facilities in resource-poor settings. The clinical validity of these indicators is assessed in a companion article. Most patients and facilities showed high levels of adherence; however, poor levels of performance in some facilities provide a target for quality improvement efforts.

## Background

Global, regional, and national health initiatives have responded to the HIV/AIDS pandemic by introducing antiretroviral therapy (ART) to ever-increasing numbers of affected patients. The success of ART relies on life-long, high levels of medication adherence to maximize clinical effectiveness and to minimize the potential population risks associated with the development of drug resistance. Evidence for a goal of maintaining adherence rates of 90% [1,2] to 95% [3] have been presented, although recent evidence has questioned the validity of that standard [4,5]. Reviews of over 50 years of research have shown that achieving medication adherence rates of over 80% for treating a range of chronic illnesses has been problematic, even in resource-rich countries [6,7]. Achieving high rates of adherence in resource poor settings is a serious challenge and performance needs to be closely monitored.

The ability to measure treatment adherence accurately is critical to identifying adherence problems and improving adherence practices. Common methods for measuring adherence to antiretroviral (ARV) medications involve using patient self-reports [8-10]; pill counts [11,12]; pharmacy dispensing data [7,13-15] and, primarily in research settings, electronic medication monitors [16-19]. Each method has strengths and limitations, but individually, each method fails to capture important dimensions of ARV treatment adherence [12,20-22]. All methods produce measurements associated with clinical outcomes in research settings; however, to our knowledge, none of the approaches to measuring adherence have been tested for feasibility and validity using routine data from the array of current treatment program settings in Africa.

Countries and global donors have agreed on the importance of supporting and monitoring adherence among patients on antiretroviral therapy [23], although they have been unable to reach consensus on optimal strategies for measuring or improving adherence at the patient or system level [23,24]. The assessment reported here was designed to investigate the feasibility of using routine data collected in a systematic way in a wide variety of facilities to construct standardized indicators that reflect the performance of facilities in maintaining their patients on therapy. In a companion article, the validity of these indicators is tested and reported [25].

In 2006, the International Network for the Rational Use of Drugs Initiative on Adherence to Antiretrovirals (INRUD-IAA), in collaboration with the national AIDS control programs from Ethiopia, Kenya, Rwanda, Tanzania, and Uganda, carried out a survey that assessed how existing ART programs and health care facilities tracked patient adherence and treatment defaulting [26]. ART programs had started in Uganda as early as 1991, in Rwanda 1999, Kenya 2001, Ethiopia 2003 and Tanzania 2004. However, the survey showed that many programs and facilities had no processes in place to measure treatment adherence or defaulting at either the patient or program level, and among those facilities that did conduct measurements, definitions and data collection practices varied widely, although they routinely collected useful data [26] .

Using these survey results along with measurement approaches described in the adherence literature [11,12], INRUD-IAA collaborators defined a draft set of core indicators for monitoring adherence and attendance that could be calculated from data routinely available in ARV treatment clinics and developed standardized methods to collect data and calculate indicators.

The draft adherence indicators and their data sources include:

• Self-reported, short-term adherence: Percentage of patients that report perfect adherence and the average percentage of doses of ARV medicine they report having missed during the previous three days, using data from interviews with patients exiting from a clinic and from self-reported adherence data recorded in clinical records.

• Dispensing-based long-term adherence: Percentage of days covered by ARV medicines dispensed over the last six months or a year and percentage of a cohort of patients who had a gap of at least 30 days between dispensing, assessed from pharmacy dispensing records.

• Consistency of visit attendance: Percentage of patients attending appointments on or before the scheduled day and percentage attending within three days after scheduled appointments, taken from clinic appointment logs.

• Pill count-based medium-term adherence: Average percentage of days patients took ARVs as expected based on pill counts as noted in clinic or pharmacy records.

## Methods

### Overall Study Design

We conducted field studies in Ethiopia, Kenya, Rwanda, and Uganda between October 2006 and June 2007 to evaluate the feasibility and the reliability of collecting data from patient interviews and medical and pharmacy records to apply to a set of draft adherence indicators for assessing adherence to ART in resource-limited settings.

### Facility Sample

In each country, the sample included 20 health facilities offering different levels of care. Eligible HIV/AIDS treatment facilities were required to have had at least 100 patients receiving ARVs a year before the study. We first stratified all such facilities in each country by region, and then chose study facilities randomly, excluding facilities that were too difficult to reach or that were the focus of other adherence-related initiatives.

### Data Collection

Trained data collectors interviewed patients exiting the facility, examined a randomly selected sample of patient records, and interviewed facility staff. Each data collection team included one team leader and three or four data collectors. Data collectors were practicing pharmacists or physicians or senior-level students. The process was designed so that one team could spend one day collecting the data in each facility.

#### Patient exit interviews

We sought to interview a purposive sample of 30 patients on ARVs as they exited from each facility following their clinic visits on the day of data collection. Data collectors first asked patients for consent and then asked them a standard question about treatment adherence over the previous three days. They also collected information on factors potentially affecting adherence, such as the time spent getting to clinic, time spent waiting in the clinic, and whether patients knew how to take their medicines correctly.

#### Retrospective sample of patient records

In each facility in Kenya and Rwanda, data collectors examined 100 randomly chosen pharmacy records from patients who had attended the clinic 13 months (Kenya) or 7 months (Rwanda) before the survey, and abstracted days of medicine each patient had been dispensed over the succeeding 12 or 6 months, respectively. In a second sample of 100 records of patients who attended the clinic 4 months prior to the day of data collection, dates of their next clinic appointment were abstracted. Data collectors abstracted pill count and self-reported adherence data from all records where available. Following a review of the experience in the first two surveys, data collectors in Uganda and Ethiopia abstracted records of a single cohort of 100 randomly selected patients in each facility who had attended the clinic 7 months before.

Additional information extracted from clinic records included demographic (age, gender) and clinical data (World Health Organization [WHO] stage at initiation, length of time on ARV therapy, and CD4 and viral load test dates and results, where available).

### Data Analysis

We entered all data into formatted Microsoft^® ^Office Excel 2003 spreadsheets and calculated the percentages and means for each indicator for all patients and for each facility, as appropriate. For each country, for each facility, we calculated the summary statistics for all patients sampled. Within country we took the median facility indicator values, as well as the maximum, 75^th ^percentile, 25^th ^percentile, and minimum. Using facility median values helped prevent skewing the summary results with outlying values, since our dual purpose was to identify individual problem facilities and to assess overall performance across facilities. For the dispensing-based indicators, we examined results for the entire sample, including patients who were lost to follow up during the observation period. The data were then entered into the Statistical Analysis System (SAS)^® ^9.1.3 and we calculated Pearson correlation coefficients between all facility-level summary indicators.

When the four pilot surveys were completed, we revised the methodology based on the overall quality of the data that facilities routinely maintain in medical and pharmacy records, the difficulty of collecting the data, the observed results, correlations among the facility-level summary indicators, and utility of the resulting information. We simplified the methodology to focus on five core adherence and attendance indicators. These five indicators were validated in a later study by comparing them to changes in clinical outcomes observed in patients newly initiating treatment; the results are reported in a companion paper [25].

### Ethics committee approval

In three of the countries the national AIDS control program gave permission to conduct the surveys and collaborated in the study as part of their quality improvement processes. In Ethiopia this approval was given by the Drug Administration and Control Authority (DACA). All patients interviewed gave verbal consent and were free to stop the interview at any time. Patient and staff interviews were conducted privately and all results were reported anonymously.

## Results

### Characteristics of Health Facilities and Patients

Characteristics of the facilities included in the sample can be seen in Table 1. Overall, we collected data from medical and pharmacy records in 78 of 80 targeted health facilities. A number of facilities did not have the target number of 30 patients attending on the day of data collection, so we interviewed a median of 21 per facility. In Ethiopia and Uganda, the median number of records examined was 100. With two samples of records in Kenya and Rwanda, the median numbers of recent (4 months before data collection) and long-term (7 or 13 months before data collection) patient records examined were 70 and 59 respectively.

**Table 1:** Characteristics of health facilities and patients included in the sample

	Kenya	Rwanda	Uganda	Ethiopia	TOTAL
**Facility Characteristics**					

# facilities	20 (19) *	20 (18,17)**	19	20	79

Teaching/Referral Hospital	7	2	5	11	25

District Hospital	6	14	7	9	36

Faith-based Hospital	5	0	3	0	8

Health Center/Clinic	1	4	2	0	7

Other Hospital	1	0	2	0	3

	**Median of facility mean**				

# of patients/week (facility minimum - maximum)	230 (48-1,525)	150 (30-750)	120 (31-2,045)	205 (40-750)	176 (30-2,045)

# of patients/hour/clinician (facility minimum - maximum)	2.2 (0.6-20)	1.9 (0.4-7.5)	2.0 (0.3-8.3)	2.0 (0.4-4.4)	2.0 (0.3-20)

**Patient Exit Interviews**					

# interviews	373	285	408	565	1,631

Age (years)	37.1	36.2	38.7	33.3	36.3

Percent female (facility minimum - maximum)	68 (38-77)	62 (47-73)	68 (46-74)	55 (14-70)	63 (14-77)

Months on treatment (facility minimum - maximum)	11 (5-34)	13 (8-27)	15 (5-27)	13 (7-20)	13 (5-34)

**Record Reviews**					

Total # of records examined	1998	2609***	1693	1982	8282

# records 13 months post ART initiation	930	1255	--	--	--

# records 4 months post ART initiation	1060	1354	--	--	--

Age (years) (facility minimum - maximum)	37 (8-43)	37 (34-40)	37 (35-41)	33 (31-38)	36 (8-43)

Percent female (facility minimum - maximum)	68 (38-77)	62 (47-73)	68 (46-74)	55 (14-70)	63 (14-77)

Months on treatment (facility minimum - maximum)	10 (3-14)	13 (8-22)	10 (4-17)	6 (2-8)	10 (2-22)

**% Patients with WHO Stage 3 or 4 at ART initiation**	--	--	79	83	81

* 20 facilities for pharmacy records, 19 for attendance and exit interviews

** 20 facilities for attendance, 18 for exit interviews, and 17 for dispensing records

*** 1255 records counted for attendance and 1279 counted for dispensing, 1154 for gap of 30 days or more in dispensing

--data not collected

### Problems in data collection

Although most data were available in the sample facilities, challenges in collecting data are outlined below.

#### Patient exit interviews

Interviewers targeted patients on ARVs except those starting ARVs on the day of the survey. In all cases the interviews were voluntary and very few patients refused. However, in some facilities, few patients attended on the day of the survey.

#### Sampling patient records

Sample patient records were usually identified by linking data from the attendance register for patients on ART in a given prior month with clinical and pharmacy records. In some cases, the attendance register did not distinguish between those on ART and those not on ART, so the sample of current patients was selected in the most feasible manner from the ART Initiation Register, from sequentially numbered clinic or pharmacy records, or from a list of all patients currently in treatment.

#### Days of medicine dispensed

In most cases, it was feasible to determine the number of days of medicines dispensed over the previous six months. However, in four facilities there were no dispensing records and the facility did not regularly prescribe the same number of days of medicine, so this indicator was not possible to measure.

### Self-Reported Adherence in Exit Interviews

**Figure 1:**
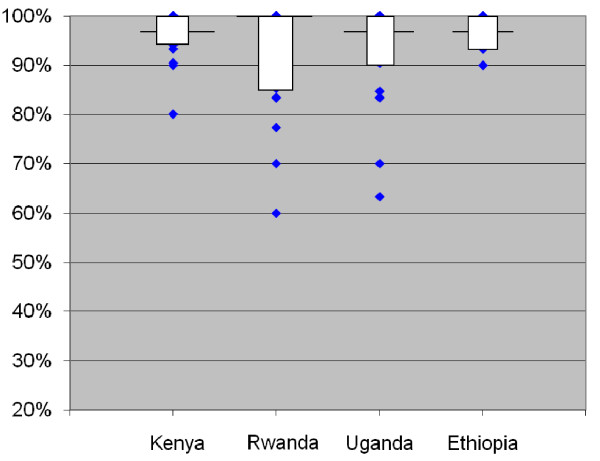
**Distribution of facility-level percentage of self-reported full adherence by country**. Note: Horizontal line represents the value of the outcome in the median facility in each country; box covers the values from the 25^th ^to 75^th ^percentile of facilities; diamonds represent values in individual facilities that lie above the 75^th ^percentile or below the 25^th ^percentile.

Ninety four percent of all patients reported perfect adherence. When analyzed by country, the percentage varied from 91% in Rwanda to 96% in Kenya and Ethiopia (Table 2), and when analyzed by facility, the median facility-specific percentage of patients with 100% self-reported adherence in the previous three days was 100% in Rwanda and 96.7% in the other three countries. However, there were facilities in which the percentage of patients with self-reported perfect adherence was as low as 60%. The overall mean percentage of doses patients reported taking across facilities was 99.4% or above; however, the facility mean was as low as 85%. (Figure 1 and Table 3).

**Table 2:** Patient indicators of adherence and clinic attendance across health facilities in four countries based on exit interviews and record review

	Kenya	Rwanda	Uganda	Ethiopia	TOTAL
*# patients interviewed*	*373*	*285*	*408*	*565*	*1631*
**% with self reported full adherence **(+/- 1.96*SE)	**96.0 (2.0)**	**90.9 (3.3)**	**92.2 (2.6)**	**96.3 (1.6)**	**94.2 (1.1)**

*# records counted for dispensed medicine*	*936*	*1279*	*1693*	*1982*	*5890*
**Percentage of days covered **(+/- 1.96*SE)	**81.6 (1.7)**	**95.1 (0.5)**	**81.8 (0.7)**	**93.0 (0.6)**	**91.1 (0.4)**

*# records counted for gap in medicine*	*936*	*1154*	*1693*	*1982*	*5765*
**% patients with gap of 30 days or more **(+/- 1.96*SE)	**25.1 (2.8)**	**4.0 (1.1)**	**18.1 (1.8)**	**10.2 (1.3)**	**13.7 (0.9)**

*# records checked for attending next appointment*	*1998*	*2609*	*1693*	*1982*	*8282*
**% attended on or before next appointment** (+/- 1.96*SE)	**78.3 (1.8)**	**78.4 (1.6)**	**71.7 (2.2)**	**73.5 (1.9)**	**75.8 (0.9)**
**% attended within 3 days of appointment **(+/- 1.96*SE)	**--**	**93.4 (1.0)**	**76.3 (2.0)**	**87.9 (0.1)**	**87.1 (0.7)**
**% attended within 30 days of appointment** (+/- 1.96*SE)	96.3 (0.8)*	**97.8 (0.6)**	**86.7 (1.6)**	**94.6 (1.0)**	**94.4 (0.5)**

-- Data not collected

* Within 60 days of appointment

**Table 3:** Facility-level indicators of adherence and clinic attendance across health facilities in four countries based on exit interviews and record review

	Median facility percentages *(minimum, 25^th ^percentile, 75^th ^percentile, maximum value)*			
**Indicator and Data Source**	**Kenya**	**Rwanda**	**Uganda**	**Ethiopia**

**Self-report from exit interview**	*(19 facilities/373 patients)*	*(18 facilities/285 patients)*	*(19 facilities/408 patients)*	*(20 facilities/565 patients)*

Perfect self-reported adherence in last 3 days	**96.7** *(80,95,100,100)*	**100** *(60,84,100,100)*	**96.7** *(63,91,100,100)*	**96.7** *(90,93,100,100)*

Mean self-reported adherence over past 3 days	**99.4** *(89,98,100,100)*	**100** *(88,95,100,100)*	**99.4** *(85,98,100,100)*	**99.6** *(96,99,100,100)*

**Days covered by dispensed medicine from records**	*(20 facilities/936 records)*	*(17 facilities/1279 records)*	*(19 facilities/1693 records)*	*(20 facilities/1982 records)*

Mean % of days covered by medicine dispensed	**90** *(25,82,95,100)*	**95** *(88,93,97,99)*	**91** *(76,86,92,97)*	**93** *(82,92,95,99)*

% of patients with ≥ 95% days covered by medicine dispensed	**63** *(12,58,79,100)*	**80** *(54,63,87,99)*	**52** *(20,46,69,81)*	**76** (55,69,82,94)

% of patients with ≥ 30 days gap in medicines dispensed	**15** *(0,7,30,41)*	**2** *(0,1,6,12)*	**19** *(0,12,24,42)*	**9** *(0,5,13,33)*

**Patient attendance at clinic from records**	*(19 facilities/1060 records)*	*(20 facilities/1354 records)*	*(19 facilities/1690 records)*	*(20 facilities/1982 records)*

On or before day of appointment	**84** *(25,77,92,100)*	**91** *(14,79,95,98)*	**79** *(15,66,87,100)*	**72** *(58,64,79,99)*

Within three days of appointment	--	**96** *(67,93,97,100)*	**80** *(20,75,91,100)*	**87** *(72,84,93,99)*

Within thirty days of appointment	98* (52,96,100,100)	**99** *(96,97,100,100)*	**95** *(29,88,97,100)*	**99** *(87,94,100,100)*

-- Data not collected

* Within 60 days of appointment

### Dispensing-Based Adherence Measures from Patient Records

Analyses of retrospective dispensing records for 5,890 patients under treatment showed that the percentage of days covered by dispensed medicines for the previous 6 months was high at 91% (95% confidence intervals (CI): 91% to 92%) with 13.7% (CI: 13%-15%) of patients having a gap of 30 days or more in dispensed medicine (Table 2).

**Figure 2:**
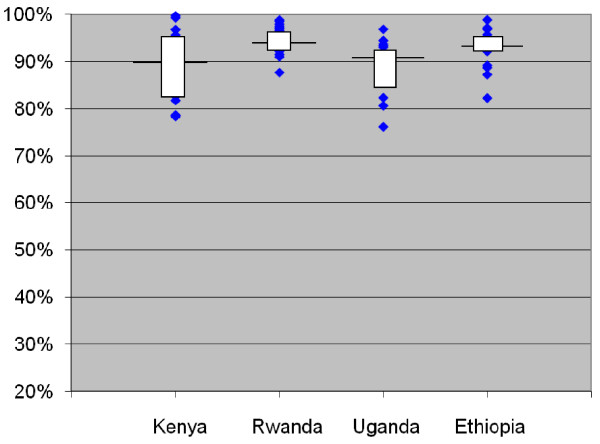
**Distribution of facility-level mean percentage of days covered by dispensed drugs in last 6 months by country**. Note: Horizontal line represents the value of the outcome in the median facility in each country; box covers the values from the 25^th ^to 75^th ^percentile of facilities; diamonds represent values in individual facilities that lie above the 75^th ^percentile or below the 25^th ^percentile.

The median facility in each country achieved a rate of days covered by dispensed medicine of 90% or above, with 2%-19% of patients experiencing a gap of 30 days or more in their medication coverage. However, the mean coverage in some individual facilities was less than 80% with as many as 42% of patients experiencing a gap of 30 days or more (Figure 2 and Table 3).

The recommended target threshold for adherence in many programs is 95%. Although the average percentage of days covered by medicine dispensed was high, fewer health facilities were able to maintain patients at this target level over the 6, or 12 month study periods. Across the four countries, the median facility-specific performance in achieving the 95% coverage target ranged from 52%-80% of patients (Table 3). In individual facilities, the percentage of patients who achieved at least 95% dispensing coverage varied from 12%-100%.

### Clinic Attendance from Clinic Records

**Figure 3:**
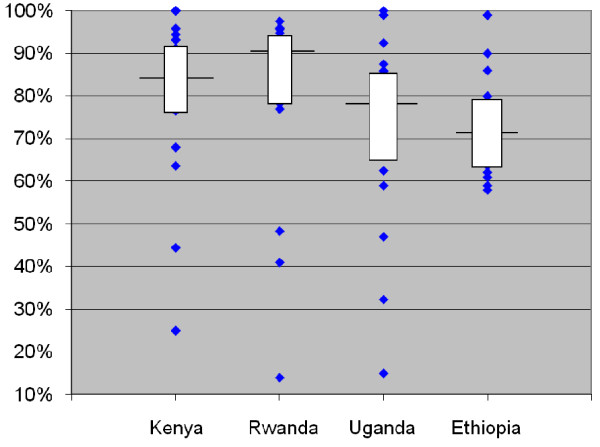
**Distribution of facility-level values for patient attendance to next appointment**. Note: Horizontal line represents the value of the outcome in the median facility in each country; box covers the values from the 25^th ^to 75^th ^percentile of facilities; diamonds represent values in individual facilities that lie above the 75^th ^percentile or below the 25^th ^percentile.

The clinic attendance measurement was lower and more variable than the medication coverage measurement. Overall, 76% (CI: 75%-77%) of patients attended their appointment on or before the scheduled day; 87% (CI: 86%-88%) attended within 3 days; and 94% (CI: 94%-95%) attended within 30 days (Table 2). Across countries, the median facility had from 72%-91% of patients attending their next appointment on or before the day scheduled and from 80%-96% attending within 3 days of their appointments (Table 3). Variability across individual facilities was larger, with only 14% of patients attending on time in one facility and 20% attending within 3 days in another facility (Figure 3).

### Pill Counts and Self-Reports in Clinic Notes

Overall, only 15% of patient records we examined included a pill count and 45% included a self-reported adherence measure; therefore, we excluded these measures.

### Correlations between Indicators

The correlations between the facility-level indicators are presented in Table 4. The correlations between the dispensing-based indicators and the other adherence indicators did not differ for the entire sample of patients (indicators 8-10 in Table 4), or for only those still in treatment at the end of the observation period (indicators 1-3). As a result we used the data from the entire sample.

**Table 4:** Correlation between facility indicators

Indicators:	2	3	4	5	6	7	8	9	10	11	12	13
**1**	**0.88753**	**-0.4332**	**0.19847**	**0.60229**	**0.24169**	0.08673	**0.78536**	**0.46213**	**-0.27041**	0.02056	0.07059	-0.00039
	<.0001	0.0001	0.0971	<.0001	0.0438	0.5946	<.0001	<.0001	0.0207	0.8629	0.5825	0.9976
	39	74	71	55	70	40	72	71	73	73	63	63

**2**	1	**-0.84969**	**0.33361**	**0.59568**	**0.50325**	**0.48017**	**0.77531**	**0.96964**	**-0.73886**	**0.48363**	0.29674	0.24588
		<.0001	0.0379	<.0001	0.0013	0.0097	<.0001	<.0001	<.0001	0.0018	0.0745	0.1424
	39	39	39	39	38	28	39	39	39	39	37	37

**3**		1	**-0.4246**	**-0.75195**	**-0.49922**	-0.05321	**-0.4183**	**-0.7567**	**0.89989**	-0.21967	-0.1382	-0.059
			0.0002	<.0001	<.0001	0.7443	0.0003	<.0001	<.0001	0.06	0.2801	0.646
		75	71	55	70	40	72	71	73	74	63	63

**4**			1	**0.74158**	**0.61924**	-0.08272	**0.41409**	**0.28721**	**-0.41749**	0.19	0.14293	0.09498
				<.0001	<.0001	0.6071	0.0003	0.0159	0.0003	0.1125	0.2718	0.4665
			73	56	72	41	71	70	72	71	61	61

**5**				1	**0.86842**	0.16057	**0.6507**	**0.61115**	**-0.71072**	**0.27723**	**0.37337**	**0.28431**
					<.0001	0.38	<.0001	<.0001	<.0001	0.0404	0.009	0.0502
				56	55	32	56	56	56	55	48	48

**6**					1	-0.02434	**0.32879**	**0.48872**	**-0.48896**	**0.26292**	**0.4468**	**0.30016**
						0.88	0.0055	<.0001	<.0001	0.0279	0.0003	0.0188
					72	41	70	69	71	70	61	61

**7**						1	0.16434	**0.36403**	-0.0598	0.08467	0.02428	0.01863
							0.3174	0.0247	0.714	0.6083	0.885	0.9116
						42	39	38	40	39	38	38

**8**							1	**0.42663**	**-0.56325**	**0.37349**	-0.08788	-0.06737
								0.0002	<.0001	0.0012	0.497	0.6029
							73	72	73	72	62	62

**9**								1	**-0.74195**	**0.4772**	0.18678	0.16058
									<.0001	<.0001	0.146	0.2125
								72	72	71	62	62

**10**									1	**-0.40181**	-0.08735	-0.06228
										0.0004	0.496	0.6277
									74	73	63	63

**11**										1	**0.26338**	**0.25041**
											0.0386	0.0496
										74	62	62

**12**											1	**0.92186**
												<.0001
											66	66

Key: In each cell

Pearson Correlation Coefficients: Bold correlations = p < 0.05

Prob > |r| under H0: Rho = 0

Number of Observations

Indicators

1. % days covered by dispensed medicine if still in treatment

2. % patients with 95% days covered by dispensed medicine if still in treatment

3. % patients with gap of 30 days or more in dispensed medicine if still in treatment

4. % patients who attended on or before their appointment

5. % patients who attended within 3 days of their appointment

6. % patients who attended within 30 days of their appointment

7. From records: % Self reported good adherence

8. % days covered by dispensed medicine

9. % patients with 95% days covered by dispensed medicine

10. % patients with gap of 30 days or more in dispensed medicine

11. % patients remaining in treatment

12. From exit interview: % full adherence

13. From exit interview: average % adherence

The percentages of days covered by dispensed medicine and of patients with more than 95% of days covered both were significantly correlated with the percentages who attended their appointments on time (i.e., on or before the scheduled day of the appointment), within 3 days, and within 30 days of their appointment, but were not correlated with self-reported adherence either in the clinical notes or in exit interviews. However, self-reported adherence at exit interview was significantly correlated with the percentage of patients who attended within 3 days of their appointment, and the percentage with perfect self-reported adherence had higher correlations with the other indicators than the average self-reported adherence. The indicator for attending appointments within 3 days had higher correlations with all indicators than the indicator for attending on or before the scheduled day of the appointment.

## Discussion

We have shown, using routine data sources, that it is possible to use standardized methods to collect data and to measure important aspects of patient treatment adherence and clinic attendance in a wide variety of health facilities in resource-poor settings and that the different facility-level summary indicator results are correlated. Because taking medicine is a private affair, all individual-level adherence measurements are indirect. Aggregated at the facility level, summary results for different indicators can point to interventions in different systems to improve facility performance.

In the four survey countries, facilities generally maintained patients at a high level of treatment adherence; however, for all indicators, we identified some facilities that performed less well, which provided a target for quality improvement efforts. The measurement approaches offer a systematic way to assess and compare adherence measures across facilities and programs and to assess the impact of system-level interventions to improve adherence.

Measuring patient self-report about recent patterns of adherence is a low cost and commonly used method to assess adherence, which has correlated with clinical outcomes both in a meta-analysis [9] and in several resource-poor settings [8,10,21,21,27], although it has been assessed in various ways [28]. For purposes of a cross-sectional survey, exit interviews provide an opportunity to standardize the self-report question (i.e., number of doses missed in the last three days; achievement of 100% adherence). In the current field studies, a small percentage of clinical records included self-reported adherence and pill counts. In addition, the self-reporting measurement in clinic records suffers from inconsistent questioning and irregular recording. Standardizing the questions and the recording methods allow for a useful comparison. Because self-report measures tend to overestimate true adherence compared to more objective measures [11,12], and the median levels reported in these surveys are over 95%, this may not be a sensitive enough measure to evaluate interventions to improve adherence.

Facilities with electronic pharmacy records often use dispensing data to calculate adherence to therapy [15]. Several studies in industrialized countries have shown associations between pharmacy-based ARV adherence measures and clinical outcomes, including viral load and CD4 counts [17,29]. These methods have also been shown to be feasible and valid in an African setting using electronic data [13]. However, using dispensing records to measure adherence in settings with manual record systems has not been widely tested. We have demonstrated that such data are possible to gather in resource-poor settings in East Africa.

The average percentage of days covered by ARV therapy dispensed over six months provides a useful patient population-based measure of intermediate-term adherence. Similarly, the percentage of patients who experience a 30-day gap in treatment measures the ability of health facilities to retain patients in consistent therapy. These measures can help a program manager identify facilities with low levels of dispensing coverage or patient retention. Different factors can contribute to low dispensing coverage and patient retention, including poor drug supply and inadequate dispensing and patient counselling practices, which can be addressed at the system level.

Patients need to attend appointments consistently to manage many clinical issues that may arise during ARV treatment and to avoid gaps in therapy. Missed appointments correlate with other aspects of non-adherence in our data and elsewhere [15,21,30,31]. Because patients often receive a 30-day supply of medicines and a follow-up appointment after 28 days, they will not miss taking their medicine, even if they do not come on their scheduled appointment date. We therefore propose attendance within three days of a scheduled appointment as a key adherence measure. Not all treatment programs in Africa have easy access to information on who is expected to attend on a given day and who has not appeared for their appointment. A standard approach for identifying patients that miss appointments could help programs develop effective community outreach systems; in addition, information on the percentage of patients missing appointments would allow a program manager to target poorly performing facilities and to identify causes and appropriate interventions.

Deciding to take steps to improve adherence levels depends on available human and financial resources. One method of deciding which facilities need to improve is to set target levels for each of the main indicators. The current results could serve as benchmarks for future surveys in other locations. Another method would be to look at the mean scores achieved by most facilities and target the poorest performing 5%, 10%, or 25% for interventions. For example, across facilities, a median of 97% of patients reported full adherence. However, in the bottom 25% of facilities, the measure was 93% or less. In the bottom 10% of facilities, 84% or less of patients reported full adherence, and in the bottom 5% of facilities, the figure fell to 78% or less (Table 5).

**Table 5:** Distribution of mean values of key adherence and attendance indicators across all countries

	Percentile	Percentage of patients with self-reported full adherence	Percentage of days covered by ARVs dispensed	Percentage of records with 30-day gap in ARVs dispensed	Percentage of patients who attended within 3 days of scheduled appointment
**Poor**	**5th**	78%	80%	37%	60%

	**10th**	84%	82%	30%	74%

	**25th**	93%	89%	19%	80%

	**Median**	97%	93%	10%	91%

	**75th**	100%	96%	4%	96%

**Good**	**90th**	100%	97%	1%	100%

Encouragingly, most facilities surveyed are maintaining high rates of patient adherence and attendance. However, all countries have facilities with low measures that need systems strengthening. Using simple, low-cost methods to identify poorly performing facilities would allow ART program managers to examine the causes of poor performance and work with facilities to make improvements. This standardized methodology and associated indicators can also be adapted to become part of an ongoing monitoring system. A manual on how to conduct the adherence assessments and spreadsheets for processing the data are available on request from the first author.

Our study made no assessment of patient retention as this was not part of our objectives. We recognize the importance of the assessment of retention. Retention data are already collected by many agencies. Our work reported here is to explore the feasibility of assessing the performance of health facilities in supporting adherence among their patients. The method of selecting patient records was to identify patients who had attended the facility 7 or 13 months before the month of the survey. To do this, the records had to be accessible in the facility. There was an implicit bias towards patients who keep appointments and those whose records were available at the facility; thus, the indicators may reflect the experience of patients still in treatment who maintain better adherence.

Evaluating and monitoring adherence to ARV treatment are critical to using health care resources effectively and efficiently, reducing rates of drug resistance to first-line therapies, and improving patient outcomes. The four field tests provide strong evidence that adherence evaluation is possible using routine data that can be collected systematically from a wide range of health facilities in resource-poor settings. The challenge now will be to introduce monitoring of these indicators on a routine basis and using them as the basis for facility-level quality improvement efforts.

## Conclusions

The four field tests have shown that data to measure indicators of adherence based on days covered by dispensed medicine, attendance at appointments and self report can be collected systematically from health facilities in a wide variety of resource-poor settings and that the indicators correlate with each other. These methods allow comparison of the performance of programs and facilities between each other, over time, and to evaluate the success of interventions.

Most facilities surveyed are maintaining high rates of patient adherence and attendance. However, all countries have facilities with low measures that need systems strengthening. Using these simple, low-cost methods to identify poorly performing facilities enables ART program managers to examine the causes of poor performance and work with facilities to make improvements.

## Competing interests

The authors declare that they have no competing interests.

## Authors' contributions

JC and DRD designed and planned the research, designed the questionnaires, analyzed the data, and drafted the article. TA, LG, JN, HT, CO and PW commented on the design of the questionnaires, selected facilities to survey in their respective countries, helped train data collectors and carried out the survey and assisted in the analysis of the data from their countries. All authors commented on article drafts and approved the final version.

## Pre-publication history

The pre-publication history for this paper can be accessed here:

http://www.biomedcentral.com/1472-6963/10/43/prepub
